# Integrated clinical and multi-omics analysis links composite inflammatory indices to macrophage-associated molecular programs in aortic dissection

**DOI:** 10.3389/fgene.2026.1840880

**Published:** 2026-05-21

**Authors:** Bo Zhang, Yanda Zhang, Rongyi Zheng, Yanwei Zhang, Long Wang

**Affiliations:** Department of Cardiothoracic Surgery, Henan Chest Hospital, Affiliated Hospital of Zhengzhou University, Zhengzhou, China

**Keywords:** aortic dissection, biomarkers, composite inflammatory indices, inflammation, machine learning, macrophages, single-cell RNA sequencing

## Abstract

**Background:**

Aortic dissection (AD) is a life-threatening vascular disease characterized by high mortality and complex pathophysiology involving inflammation and vascular wall degeneration. Composite inflammatory indices derived from routine blood tests have shown prognostic value in AD; however, their role in disease assessment and their integration with molecular mechanisms remain unclear.

**Methods:**

A total of 288 participants (144 AD patients and 144 controls) were enrolled for clinical analysis. Six composite inflammatory indices (NLR, MLR, SIRI, SII, PHR, and AISI) were calculated and evaluated using logistic regression and restricted cubic spline (RCS) models. Generalized propensity score (GPS) weighting was applied to reduce confounding. Multi-omics analyses were conducted by integrating single-cell RNA sequencing (GSE213740), bulk transcriptomic data (GSE153434), and inflammation-related gene sets. Weighted gene co-expression network analysis (WGCNA) and differential expression analysis were performed to identify candidate genes. Machine learning algorithms (LASSO, random forest, and SVM-RFE) were used for feature selection. External validation (GSE52093) and RT-qPCR experiments were conducted to verify feature genes.

**Results:**

All six inflammatory indices were significantly elevated in AD patients (all P < 0.001) and independently associated with AD. RCS analysis revealed distinct nonlinear patterns, with AISI and SII showing continuous dose–response relationships, whereas NLR, MLR, and SIRI exhibited threshold effects. Associations remained robust across subgroups and after GPS weighting, with improved covariate balance (correlation coefficients <0.1). Single-cell analysis identified macrophages as the most prominently altered cell population, with 1,063 differentially expressed genes indicating extensive transcriptional reprogramming. Bulk RNA-seq analysis identified 2,766 DEGs (1,331 upregulated and 1,435 downregulated), and WGCNA revealed a key module (2,551 genes) strongly associated with AD (r = 0.98). Integrative analysis yielded 25 candidate genes, from which four genes (HIF1A, ITGA5, PLAUR, and TLR2) were consistently selected by machine learning. External validation and RT-qPCR confirmed significant upregulation of HIF1A, ITGA5, and PLAUR in AD tissues.

**Conclusion:**

Composite inflammatory indices are strongly associated with AD risk, and inflammatory-associated genes, particularly HIF1A, ITGA5, and PLAUR, may serve as potential diagnostic biomarkers and mechanistic targets.

## 1 Introduction

Aortic dissection (AD) is a life-threatening cardiovascular emergency caused by a tear in the aortic intima, allowing blood to penetrate the aortic wall and propagate within the media to create a false lumen (Bossone and Eagle, 2021). Acute aortic dissection of the ascending aorta is highly lethal in untreated symptomatic patients, with an early mortality rate of approximately 1% per hour after symptom onset (Harris et al., 2022). Patients with uncomplicated acute type B aortic dissection have a 30-day mortality rate of approximately 10%, whereas those who develop malperfusion or rupture face a mortality rate reaching 25% by day 30 (Smedberg et al., 2024). Globally, aortic dissection imposes a substantial clinical burden. Population-based analyses across numerous countries demonstrate a pooled incidence of approximately 4.8 per 100,000 individuals per year, with aortic dissection remaining a highly fatal disease with limited predictability, necessitating an emergency response infrastructure and representing a major public health challenge (Gouveia et al., 2022). Despite advances in imaging and surgical management, timely diagnosis remains challenging because the clinical presentation overlaps with other acute cardiovascular conditions, and reliable circulating biomarkers for early risk stratification are still lacking (Hanna et al., 2025).

The pathogenesis of AD involves the interplay of structural aortic wall degeneration and a robust inflammatory response (Wang et al., 2020). Inflammation is now recognized as both a trigger and a propagating mechanism of aortic dissection, contributing to extracellular matrix degradation, vascular smooth muscle cell apoptosis, and progressive medial destruction (He et al., 2025; Gryka-marton et al., 2024). Among circulating indicators of systemic inflammation, composite hematological indices derived from routine blood counts have attracted growing interest. These indices, including the neutrophil-to-lymphocyte ratio (NLR), monocyte-to-lymphocyte ratio (MLR), systemic immune-inflammation index (SII), systemic inflammatory response index (SIRI), platelet-to-HDL-cholesterol ratio (PHR), and aggregate index of systemic inflammation (AISI), reflect the balance between pro-inflammatory and anti-inflammatory cellular components and are readily obtainable at low cost (Xin et al., 2025; Van Damme et al., 2025). The NLR, MLR, and SII have been specifically investigated in the context of acute aortic dissection, with elevated levels of these indices found to be associated with an increased risk of in-hospital all-cause mortality (Ustaalioglu and Aydogdu, 2024). The SII, which more comprehensively reflects the balance between host inflammatory and immune status, has been examined as a predictive factor for adverse aortic-related events following endovascular repair in type B dissection (Su et al., 2022; Zhao et al., 2022). Although these associations have been described in outcome-focused studies, systematic evidence characterizing the associations between multiple composite inflammatory indices and AD at the time of clinical presentation, particularly in combination with molecular analyses, remains limited.

The six indices were selected based on three criteria: biological complementarity, prior evidence in vascular or aortic disease, and availability from routine admission tests. Despite partial overlap in cellular components, each index reflects a distinct aspect of systemic inflammation. NLR represents the balance between innate (neutrophil) and adaptive (lymphocyte) immunity and is widely associated with outcomes in acute aortic dissection. MLR emphasizes monocyte-driven inflammation, relevant given the central role of macrophage infiltration in aortic wall injury. SIRI combines neutrophil and monocyte signals, providing a broader measure of innate immune activation. SII incorporates platelets, capturing thromboinflammatory processes linked to endothelial injury and dissection progression. PHR uniquely integrates HDL-C, reflecting lipid-inflammation interplay. AISI includes all major cellular components, offering a more global assessment of systemic inflammation. Together, these indices cover complementary domains-immune balance, thromboinflammation, and lipid-related inflammation-rather than redundant signals.

At the cellular level, macrophages have emerged as key effectors within the aortic inflammatory microenvironment. Infiltrating macrophages in the aortic wall secrete inflammatory cytokines and matrix metalloproteinases that contribute to extracellular matrix degradation, thereby playing an important role in the pathogenesis of aortic dissection (Liu et al., 2022). Single-cell RNA sequencing studies of human acute aortic dissection samples have demonstrated that IL-1β-expressing inflammatory macrophages and classical monocytes are markedly increased, with trajectory analyses confirming that these inflammatory macrophages differentiate from S100A8/9/12-positive classical monocytes uniquely observed in the dissected aorta (Inoue et al., 2024). scRNA-seq analysis of ascending aortic wall tissue has further revealed that the proportion of macrophages is significantly higher in acute type A aortic dissection tissues than in normal controls, with extensive transcriptional reprogramming confirmed including upregulation of matrix metalloproteinases MMP2 and MMP9 (Zhang et al., 2023). Together, these findings underscore the pivotal role of macrophage-mediated inflammation in disease initiation and progression, although the specific inflammation-related genes driving this process have not been fully characterized (Li S. et al., 2024).

Recent transcriptomic studies have sought to identify molecular signatures of AD using integrative approaches. Weighted gene co-expression network analysis (WGCNA) and differential expression analysis of publicly available datasets have revealed gene modules strongly correlated with disease status, while machine learning algorithms offer a complementary framework for selecting robust biomarkers from large candidate gene lists (Feng et al., 2023; Hou et al., 2025). Among genes emerging from these analyses, HIF1A, ITGA5, PLAUR, and TLR2 have been repeatedly implicated. HIF1A has been shown to transcriptionally regulate multiple downstream target genes relevant to inflammation, including TLR2, as well as genes involved in glycolysis and tissue remodeling, and its expression is significantly upregulated in macrophages from patients with aortic dissection (Li HB. et al., 2024). WGCNA-based analyses have identified ITGA5 and PLAUR among the top genes in aortic dissection, with candidate genes enriched in pathways related to hypoxia, inflammation, cell death, and extracellular matrix regulation (Feng et al., 2023). These candidates have shown consistent differential expression across independent transcriptomic datasets, yet experimental validation in clinical tissue samples and integration with composite inflammatory indices remain underexplored.

Given these gaps, the present study was designed to conduct a combined clinical and multi-omics investigation to characterize the relationship between composite inflammatory indices and AD risk, identify macrophage-driven inflammation-related genes through integrative bioinformatics and machine learning analyses, and validate key molecular targets at the transcriptomic and experimental levels. By linking systemic inflammatory biomarkers with cellular and molecular mechanisms, this work aims to provide a more comprehensive understanding of the inflammatory basis of AD and to identify candidates suitable for diagnostic application.

## 2 Materials and methods

### 2.1 Study population and data collection

This study was conducted as a combined clinical and multi-omics investigation to identify inflammation-related biomarkers associated with aortic dissection (AD). A total of 288 participants were consecutively enrolled from Henan Provincial Chest Hospital, including 144 patients diagnosed with AD and 144 non-AD controls. The non-AD controls were healthy individuals identified through routine physical examination at the same institution during the study period. Inclusion criteria for controls included: (1) age ≥18 years; (2) no history or imaging evidence of aortic dissection or acute aortic syndrome; (3) absence of active infection, autoimmune disease, malignancy, or hematological disorders known to independently influence systemic inflammatory indices; and (4) no established diagnosis of major acute cardiovascular conditions, including acute myocardial infarction or decompensated heart failure. Individuals who did not meet all inclusion criteria were excluded from the control group. The diagnosis of AD was confirmed by computed tomography angiography or magnetic resonance angiography according to established clinical guidelines. Demographic characteristics, laboratory parameters, comorbidities (including hypertension, diabetes mellitus, and hyperlipidemia), and lifestyle factors such as smoking and alcohol consumption were collected from electronic medical records. The baseline characteristics of the study population are presented in Table 1. This study was approved by the Ethics Committee of Henan Provincial Chest Hospital and conducted in accordance with the Declaration of Helsinki. Written informed consent was obtained from all participants or their legal representatives.

**TABLE 1:** Baseline clinical characteristics of AD patients.

Characteristics	Total (n = 288)	AD (n = 144)	Non-AD (n = 144)	P value
Age, mean (SD)	63.4 (12.0)	68.0 (11.0)	58.8 (11.3)	<0.001
Sex (n, %)	​	​	​	<0.001
Male	204 (70.8)	117 (81.2)	87 (60.4)	​
Female	84 (29.2)	27 (18.8)	57 (39.6)	​
Smoking (%)	124 (43.1)	86 (59.7)	38 (26.4)	<0.001
Alcoholic (%)	96 (33.3)	61 (42.4)	35 (24.3)	0.002
Hypertension (%)	130 (45.1)	96 (66.7)	34 (23.6)	<0.001
Hyperlipidemia (%)	130 (45.1)	90 (62.5)	40 (27.8)	<0.001
DM (%)	100 (34.7)	68 (47.2)	32 (22.2)	<0.001

### 2.2 Calculation of composite inflammatory indices

Peripheral blood samples collected at admission were used to calculate six composite inflammatory indices. The neutrophil-to-lymphocyte ratio (NLR) was defined as neutrophil count divided by lymphocyte count, and the monocyte-to-lymphocyte ratio (MLR) as monocyte count divided by lymphocyte count. The systemic inflammatory response index (SIRI) was calculated as neutrophil count multiplied by monocyte count divided by lymphocyte count, whereas the systemic immune-inflammation index (SII) was defined as platelet count multiplied by neutrophil count divided by lymphocyte count. The platelet-to-high-density lipoprotein ratio (PHR) was calculated as platelet count divided by HDL-C concentration, and the aggregate index of systemic inflammation (AISI) was defined as platelet count multiplied by neutrophil count and monocyte count divided by lymphocyte count. All hematological parameters were measured using automated analyzers, and HDL-C levels were determined using standardized enzymatic assays.

### 2.3 Association analysis of inflammatory indices with AD risk

Logistic regression models were constructed to evaluate the association between composite inflammatory indices and the risk of aortic dissection, with results presented as odds ratios (ORs) and 95% confidence intervals (CIs). Each inflammatory index was analyzed as a continuous variable. Restricted cubic spline (RCS) regression was further applied to characterize potential nonlinear associations by modeling ORs across the continuous range of inflammatory indices. Subgroup analyses were performed stratified by clinical variables, and the corresponding effect estimates were presented as forest plots. Interaction effects were assessed to evaluate potential effect modification across subgroups.

### 2.4 Generalized propensity score analysis

To further reduce potential confounding, generalized propensity score (GPS) weighting based on generalized linear models was applied. The GPS model was constructed by regressing each composite inflammatory index (treated as a continuous exposure variable) on the following pre-specified baseline covariates: age, sex, smoking status, alcohol consumption, hypertension, diabetes mellitus, and hyperlipidemia. These covariates were selected *a priori* based on their established clinical relevance as potential confounders of the relationship between systemic inflammation and aortic dissection. A generalized linear model with a Gaussian distribution and identity link was used to estimate the GPS for each participant. Stabilized inverse probability weights were subsequently calculated to balance the distribution of covariates across levels of each inflammatory index. Covariate balance before and after weighting was evaluated using absolute correlation coefficients between each covariate and the inflammatory index of interest, with values less than 0.1 considered indicative of adequate balance. Weighted logistic regression models were then applied to reassess the associations between inflammatory indices and AD as a sensitivity analysis.

### 2.5 Single-cell RNA sequencing data processing

Single-cell RNA sequencing data were obtained from the GSE213740 dataset, including six AD samples and three control samples. Raw 10× Genomics data were processed using the Seurat package. After gene annotation, individual samples were merged into a single dataset for downstream analysis. Quality control was performed by excluding cells with fewer than 200 or more than 5,000 detected genes, fewer than 500 or more than 40,000 UMIs, log10GenesPerUMI <0.8, or mitochondrial gene proportion >20%. Samples with low cell numbers after filtering were removed. Data were normalized, and the top 2,000 highly variable genes were identified for subsequent analysis after excluding non-coding genes, pseudogenes, and mitochondrial genes. Dimensionality reduction was performed using principal component analysis, followed by visualization with t-distributed stochastic neighbor embedding and Uniform Manifold Approximation and Projection. Batch effects across samples were corrected using the Harmony algorithm, and clustering was performed using a graph-based approach, with the resolution determined based on clustering stability and marker gene expression patterns.

Cell-type annotation was conducted based on canonical marker genes derived from previous studies (Guo et al., 2026), including MYH11, ACTA2, and CNN1 for smooth muscle cells, PDGFRA, DCN, and LUM for fibroblasts, ITGAM and CD14 for macrophages, CD3D and CD3G for T cells, CD79A and CD19 for B cells, and CDH5 and TIE1 for endothelial cells. Comparative analysis revealed that macrophages were the most prominently altered cell population between AD and control samples, and macrophage marker genes were extracted for subsequent integrative analyses.

### 2.6 Differential expression analysis

To identify genes associated with aortic dissection at the transcriptomic level, bulk RNA sequencing data from the GSE153434 dataset, including 10 AD samples and 10 control samples, were analyzed. Gene expression data were normalized, and differential expression analysis was performed using the limma package. Genes with an absolute log2 fold change greater than 1 and an adjusted P value less than 0.05 were considered differentially expressed genes (DEGs).

### 2.7 Weighted gene co-expression network analysis

Weighted gene co-expression network analysis was performed to identify gene modules associated with aortic dissection. To reduce noise and improve computational efficiency, the top 5000 genes ranked by median absolute deviation were selected for network construction. Sample quality was assessed using hierarchical clustering, and outlier samples were removed based on a predefined cut height. A soft-thresholding power was determined according to the scale-free topology criterion by evaluating a series of candidate powers, and the optimal value was selected when the scale-free topology fit index reached a stable plateau. The adjacency matrix was constructed based on pairwise gene correlations and subsequently transformed into a topological overlap matrix, which was further converted into a dissimilarity matrix for hierarchical clustering. Gene modules were identified using dynamic tree cutting with a minimum module size of 50 genes, and modules with high similarity were merged according to module eigengene correlations. Module eigengenes were then calculated and correlated with disease status to identify clinically relevant modules, and the relationships between modules and traits were visualized using heatmaps, with additional assessment of network structure based on eigengene adjacency and topological overlap plots. The module showing the strongest association with aortic dissection was selected as the key module, and genes within this module were extracted for downstream analyses.

### 2.8 Identification of key inflammation-related genes

To identify key inflammation-related genes associated with aortic dissection, an integrative analysis was performed by combining multiple levels of evidence. Inflammation-related genes were obtained from the Molecular Signatures Database (MSigDB) based on Hallmark gene sets, and these genes were intersected with differentially expressed genes identified from bulk transcriptomic analysis, genes within the disease-associated module identified by weighted gene co-expression network analysis, and macrophage marker genes derived from single-cell RNA sequencing. The overlapping genes were considered candidate inflammation-related genes for further analysis, and the results of the intersection were visualized using a Venn diagram.

### 2.9 Machine learning-based feature selection

To further refine candidate genes, three machine learning algorithms were applied for feature selection (Guo et al., 2026), including least absolute shrinkage and selection operator (LASSO), random forest (RF), and support vector machine–recursive feature elimination (SVM-RFE). For LASSO analysis, a binomial logistic regression model was constructed using the glmnet package, and the optimal penalty parameter was determined by ten-fold cross-validation, with genes corresponding to nonzero coefficients retained. For the random forest model, feature importance was evaluated based on the mean decrease in Gini index, and the top-ranking genes were selected. For SVM-RFE, recursive feature elimination with cross-validation was performed to identify the optimal subset of genes that achieved the highest classification accuracy. Genes consistently selected across all three algorithms were defined as feature genes and retained for subsequent analyses.

### 2.10 External transcriptomic validation

To further evaluate the robustness of the identified feature genes, external validation was performed using an independent transcriptomic dataset (GSE52093), which included seven aortic dissection samples and five control samples. The expression levels of candidate genes were compared between groups to assess the consistency of findings across datasets.

### 2.11 RT-qPCR experimental verification

To further validate gene expression at the experimental level, aortic tissue samples were collected from 10 individuals at Henan Provincial Chest Hospital, including five patients with aortic dissection and five control samples obtained from normal vascular tissues during coronary artery bypass grafting. All tissue specimens were obtained intraoperatively and immediately rinsed with sterile saline to remove blood contamination. Samples were then snap-frozen in liquid nitrogen and stored at −80 °C until further processing. Total RNA was extracted from tissue samples using standard protocols, and RNA concentration and purity were assessed prior to downstream analysis. Complementary DNA was synthesized through reverse transcription using a commercially available kit. Gene expression levels were quantified by real-time quantitative polymerase chain reaction (RT-qPCR) using gene-specific primers and a SYBR Green detection system on a quantitative PCR platform. Relative expression levels were calculated using the 2^-ΔΔCt method with an internal reference gene for normalization. All reactions were performed in triplicate, and mean values were used for subsequent analysis to ensure reproducibility and reliability.

### 2.12 Statistical analysis

All statistical analyses were performed using R software (version 4.4.2). Continuous variables were expressed as mean ± standard deviation and compared using Student’s t-test or the Mann-Whitney U test as appropriate, while categorical variables were presented as counts (percentages) and compared using the chi-square test. Logistic regression models were used to estimate odds ratios (ORs) and 95% confidence intervals (CIs) for the association between inflammatory indices and aortic dissection. Restricted cubic spline regression was applied to explore potential nonlinear relationships. Generalized propensity score weighting was used to reduce confounding, and covariate balance was assessed using correlation coefficients. For transcriptomic analyses, differential expression was performed using the limma package, and machine learning models were implemented using glmnet, randomForest, and caret packages. RT-qPCR data were analyzed and visualized using GraphPad Prism (version 9). A two-sided P value < 0.05 was considered statistically significant.

## 3 Results

### 3.1 Elevated inflammatory indices in aortic dissection

A total of 288 participants were included, comprising 144 patients with aortic dissection and 144 controls (Table 1). Patients with aortic dissection were significantly older than controls (68.0 ± 11.90 vs. 58.8 ± 11.3 years, P < 0.001) and had a higher proportion of males (81.2% vs. 60.4%, P < 0.001). The prevalence of smoking (59.7% vs. 26.4%, P < 0.001), alcohol consumption (42.4% vs. 24.3%, P = 0.002), hypertension (66.7% vs. 23.6%, P < 0.001), hyperlipidemia (62.5% vs. 27.8%, P < 0.001), and diabetes mellitus (47.2% vs. 22.2%, P < 0.001) was significantly higher in the aortic dissection group.

Consistent with these findings, all six composite inflammatory indices were significantly elevated in patients with aortic dissection compared with controls (Table 2), including NLR (4.0 ± 2.5 vs. 2.6 ± 1.7), MLR (0.4 ± 0.2 vs. 0.3 ± 0.1), SIRI (2.4 ± 2.1 vs. 1.2 ± 1.1), SII (976.9 ± 838.4 vs. 515.6 ± 319. 1), PHR (258.7 ± 121.0 vs. 191.6 ± 75.0), and AISI (611.2 ± 642.6 vs. 243.9 ± 219.3) (all P < 0.001). Notably, indices integrating multiple inflammatory cell components, particularly SIRI, SII, and AISI, showed more pronounced elevations, indicating a markedly enhanced systemic inflammatory response in aortic dissection.

**TABLE 2:** Comparison of six inflammatory factor complexes in the two groups of patients.

Characteristics	Total (n = 288)	AD (n = 144)	Non-AD (n = 144)	P value
NLR	3.3 (2.2)	4.0 (2.5)	2.6 (1.7)	<0.001
MLR	0.4 (0.2)	0.4 (0.2)	0.3 (0.1)	<0.001
SIRI	1.8 (1.8)	2.4 (2.1)	1.2 (1.1)	<0.001
SII	746.2 (674.1)	976.9 (838.4)	515.5 (319.1)	<0.001
PHR	225.1 (105.9)	258.7 (121.0)	191.6 (75.0)	<0.001
AISI	427.5 (513.3)	611.2 (642.6)	243.9 (219.3)	<0.001

### 3.2 Inflammatory indices are positively associated with aortic dissection

**FIGURE 1:**
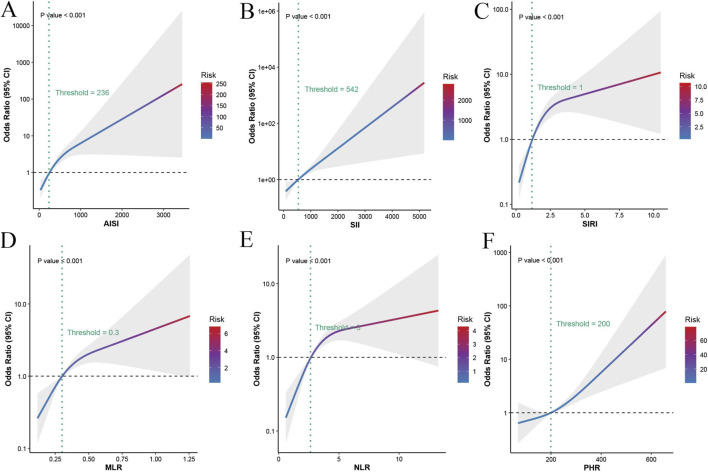
Association of six composite inflammatory indices with the risk of aortic dissection. Nonlinear associations between AISI **(A)**, SII **(B)**, SIRI **(C)**, MLR **(D)**, NLR **(E)**, and PHR **(F)** and AD risk are shown. The solid lines represent odds ratios (ORs), and shaded areas indicate 95% confidence intervals (CIs). The horizontal dashed line represents OR = 1. Vertical dashed lines indicate estimated threshold values.

All six composite inflammatory indices were significantly associated with an increased risk of aortic dissection (all P < 0.001, Figure 1). As illustrated in Figure 1, the odds of aortic dissection increased progressively with higher levels of inflammatory indices, although distinct nonlinear patterns were observed across different markers. Specifically, AISI and SII demonstrated a continuous and steady increase in risk across their entire range (Figures 1A,B), indicating a strong exposure-odds relationship. In contrast, SIRI, NLR, and MLR exhibited a rapid increase in risk at lower levels followed by a more gradual rise (Figures 1C–E), suggesting the presence of threshold effects. Correspondingly, inflection points were identified at approximately 236 for AISI, 542 for SII, 1 for SIRI, three for NLR, and 0.3 for MLR. PHR also showed a positive association with aortic dissection risk (Figure 1F), with a relatively modest increase at lower levels and a more pronounced rise beyond the threshold of approximately 200. Overall, these findings indicate that elevated inflammatory indices are strongly associated with aortic dissection, with evident nonlinear dose–response relationships.

### 3.3 Consistent associations across subgroups

**FIGURE 2:**
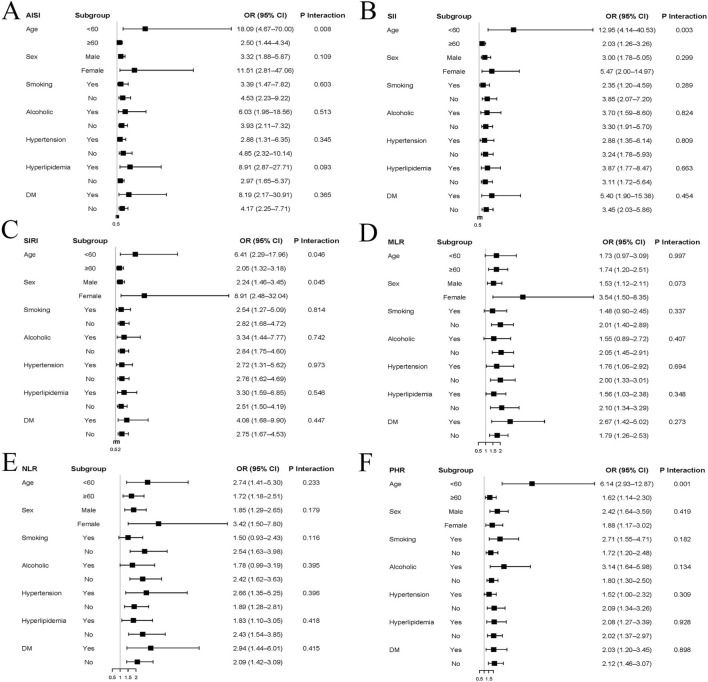
Subgroup analyses of the associations between inflammatory indices and aortic dissection risk. Forest plots showing odds ratios (ORs) and 95% confidence intervals (CIs) for the associations between AISI **(A)**, SII **(B)**, SIRI **(C)**, MLR **(D)**, NLR **(E)**, and PHR **(F)** and AD risk across predefined subgroups, including age, sex, smoking status, alcohol consumption, hypertension, hyperlipidemia, and diabetes mellitus.

Subgroup analyses demonstrated that the associations between inflammatory indices and aortic dissection remained generally consistent across different clinical subgroups (Figures 2A–F). Elevated levels of all six inflammatory indices were associated with higher odds of aortic dissection in most subgroups, including stratifications by age, sex, smoking status, alcohol consumption, hypertension, hyperlipidemia, and diabetes mellitus. Notably, stronger associations were observed in younger individuals (<60 years) compared with older patients for several indices, particularly AISI, SII, and PHR, with significant interaction effects detected (P for interaction < 0.05). In addition, sex-specific differences were observed for SIRI, with a more pronounced association in females than in males. Despite these variations, the overall direction of association remained consistent across all subgroups. For most other stratified analyses, no significant interaction effects were identified, indicating that the associations between inflammatory indices and aortic dissection were largely stable and not substantially modified by conventional clinical risk factors. These findings further support the robustness and generalizability of inflammatory indices as risk-related markers for aortic dissection.

### 3.4 Improved covariate balance after GPS weighting

**FIGURE 3:**
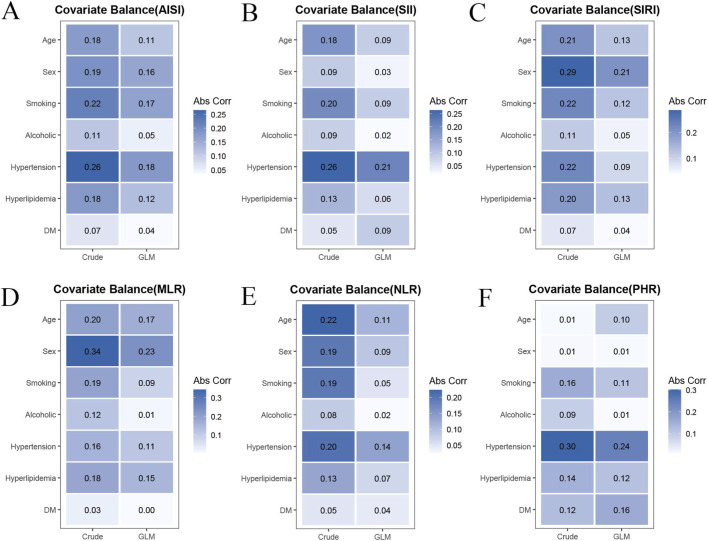
Covariate balance before and after generalized propensity score (GPS) weighting. Heatmaps showing absolute correlation coefficients between inflammatory indices and baseline covariates before (Crude) and after weighting (GLM-based GPS). Corresponding results for AISI **(A)**, SII **(B)**, SIRI **(C)**, MLR **(D)**, NLR **(E)**, and PHR **(F)**, respectively. Color intensity represents the magnitude of correlation.

Generalized propensity score weighting markedly improved covariate balance across all inflammatory indices (Figure 3). For AISI, the absolute correlation coefficients between inflammatory indices and baseline covariates were noticeably reduced after weighting (Figure 3A). Similar improvements were observed for SII and SIRI, with most covariates showing substantially lower correlations in the weighted models compared with crude analyses (Figures 3B,C). For MLR and NLR, covariate imbalance was also effectively attenuated following GPS weighting, particularly for variables such as sex and smoking (Figures 3D,E). Likewise, PHR demonstrated improved balance across most covariates after weighting, with overall reductions in correlation coefficients (Figure 3F). Across all indices, the majority of covariates approached or fell below the threshold of 0.1 after weighting, indicating adequate balance. These findings suggest that GPS weighting effectively minimized baseline differences between groups and enhanced the robustness of the observed associations between inflammatory indices and aortic dissection.

### 3.5 Macrophages are prominently altered in aortic dissection

**FIGURE 4:**
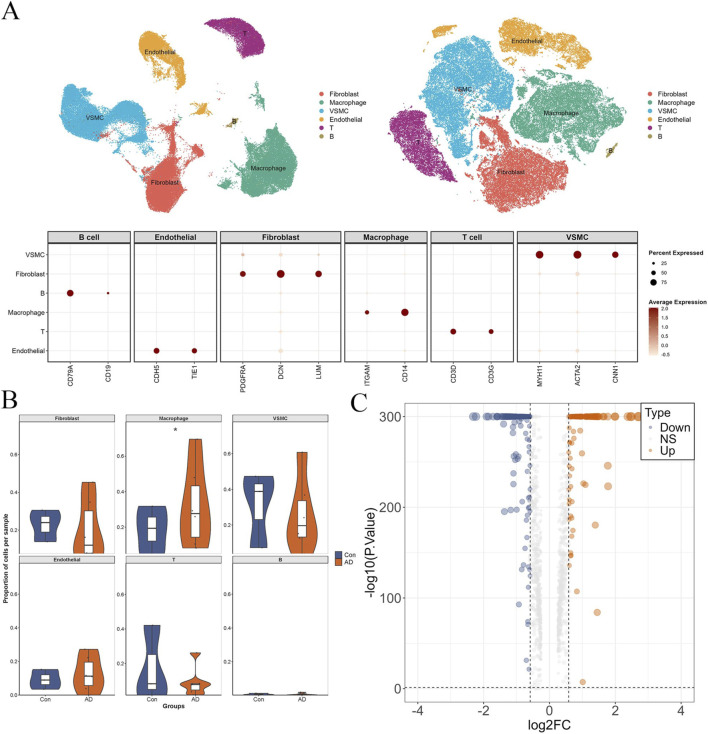
Single-cell transcriptomic landscape of aortic dissection. **(A)** UMAP visualization of major cell populations identified in control and AD samples. **(B)** Comparison of cell-type proportions between AD and control groups. **(C)** Volcano plot of differentially expressed genes in macrophages between AD and control groups.

To further investigate the cellular basis of the inflammatory alterations observed in aortic dissection, we performed single-cell RNA sequencing analysis on nine samples, including six aortic dissection samples and three control samples. As shown in Figure 4A, six major cell populations were identified, including vascular smooth muscle cells (VSMCs), fibroblasts, endothelial cells, macrophages, T cells, and B cells, based on canonical marker gene expression.

Analysis of cellular composition revealed evident differences between the aortic dissection and control groups. Among all identified cell populations, macrophages showed the most pronounced change, with a significantly increased proportion in aortic dissection samples compared with controls (Figure 4B). In contrast, the proportions of other cell types exhibited relatively limited or less consistent alterations. These results indicate that macrophages may be one of the most prominently altered cell populations during aortic dissection. To further define transcriptional alterations in macrophages, differential expression analysis was performed between macrophages derived from aortic dissection and control samples. As shown in Figure 4C, a total of 1,063 differentially expressed genes were identified in macrophages, suggesting extensive transcriptional reprogramming of this cell population in aortic dissection. Together, these findings support a potentially important role for macrophages in the inflammatory microenvironment of aortic dissection.

### 3.6 Identification of key modules by WGCNA in bulk transcriptomic data

**FIGURE 5:**
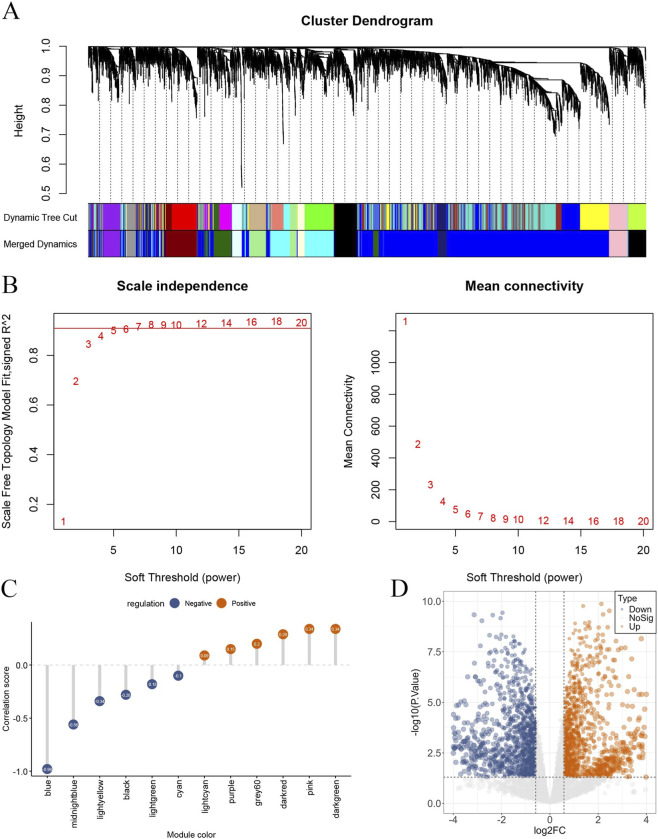
Identification of key gene modules and differentially expressed genes in bulk transcriptomic data. **(A)** Hierarchical clustering dendrogram showing gene modules identified by dynamic tree cutting. **(B)** Determination of the soft-thresholding power based on scale-free topology fit index and mean connectivity. **(C)** Module-trait relationship analysis demonstrating correlations between gene modules and AD status. **(D)** Volcano plot of differentially expressed genes (DEGs) between AD and control samples.

To further validate the transcriptional alterations at the bulk level, weighted gene co-expression network analysis (WGCNA) was performed using the GSE153434 dataset, including 20 samples (10 aortic dissection and 10 control samples). As shown in Figure 5A, hierarchical clustering identified gene modules based on topological overlap, and similar modules were merged according to dynamic tree cutting. The soft-thresholding power was determined to be β = 8, which satisfied the scale-free topology criterion (Figure 5B). Module–trait relationship analysis demonstrated that the blue module exhibited the strongest correlation with aortic dissection (r = 0.98; Figure 5C). This module contained a total of 2,551 genes, suggesting that it may represent a key co-expression network associated with disease progression.

In parallel, differential expression analysis was conducted based on the bulk transcriptomic data. As shown in Figure 5D, a total of 2,766 differentially expressed genes (DEGs) were identified, including 1,331 upregulated genes and 1,435 downregulated genes in aortic dissection compared with controls.

### 3.7 Identification of candidate genes by integrative analysis

**FIGURE 6:**
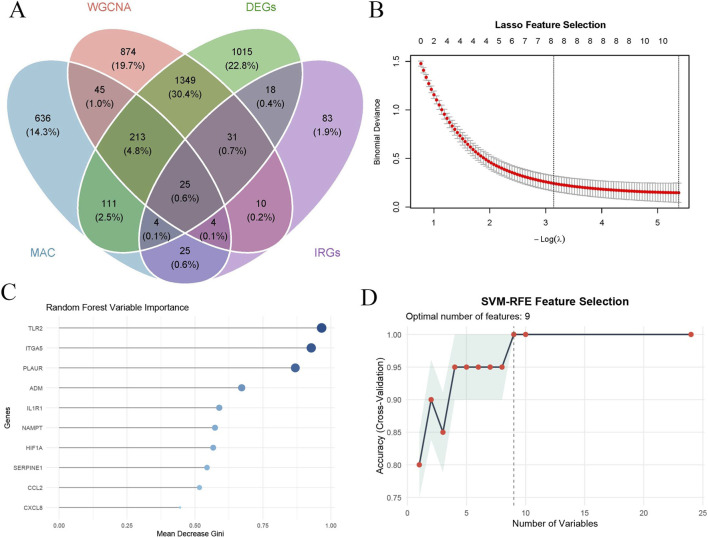
Identification of candidate and key machine learning-selected genes associated with aortic dissection. **(A)** Venn diagram showing the intersection of macrophage-derived genes, bulk DEGs, WGCNA module genes, and inflammation-related genes. **(B)** LASSO regression analysis for feature selection with optimal lambda determined by cross-validation. **(C)** Random forest analysis showing gene importance ranked by mean decrease Gini index. **(D)** SVM-RFE analysis identifying the optimal number of features based on classification accuracy.

To further refine candidate genes associated with aortic dissection, an integrative analysis was performed by combining multiple gene sets. Inflammatory response-related genes were obtained from the MSigDB database, yielding a total of 201 inflammatory genes. As shown in Figure 6A, four gene sets were included in the intersection analysis: macrophage-derived differentially expressed genes, bulk RNA-seq DEGs, WGCNA blue module genes, and inflammatory genes. Venn analysis identified 25 overlapping genes shared across all four datasets, which were defined as candidate genes for subsequent machine learning analysis.

### 3.8 Identification of key candidate genes by machine learning algorithms

To further refine the candidate genes and identify robust biomarkers associated with aortic dissection, three machine learning algorithms, including least absolute shrinkage and selection operator (LASSO), random forest (RF), and support vector machine recursive feature elimination (SVM-RFE), were applied to the 25 candidate genes obtained from integrative analysis. LASSO regression analysis identified a total of 8 genes under the optimal penalty parameter (Figure 6B), including ADM, CD55, CD82, HBEGF, HIF1A, ITGA5, PLAUR, and TLR2. Random forest analysis selected 10 genes with the highest importance scores based on the mean decrease Gini index (Figure 6C), including TLR2, ITGA5, PLAUR, ADM, IL1R1, NAMPT, HIF1A, SERPINE1, CCL2, and CXCL8. SVM-RFE analysis further identified 9 genes that achieved the highest classification accuracy (Figure 6D), including ITGA5, PLAUR, TLR2, IL1R1, NAMPT, HIF1A, FPR1, CD82, and CXCL8. By intersecting the gene sets obtained from the three machine learning algorithms, four overlapping genes, namely, HIF1A, ITGA5, PLAUR, and TLR2, were ultimately identified as key candidate genes. These genes were consistently selected across different analytical approaches, suggesting their strong stability and potential key roles in the molecular mechanisms underlying aortic dissection.

### 3.9 Validation of key candidate genes in independent datasets and clinical samples

**FIGURE 7:**
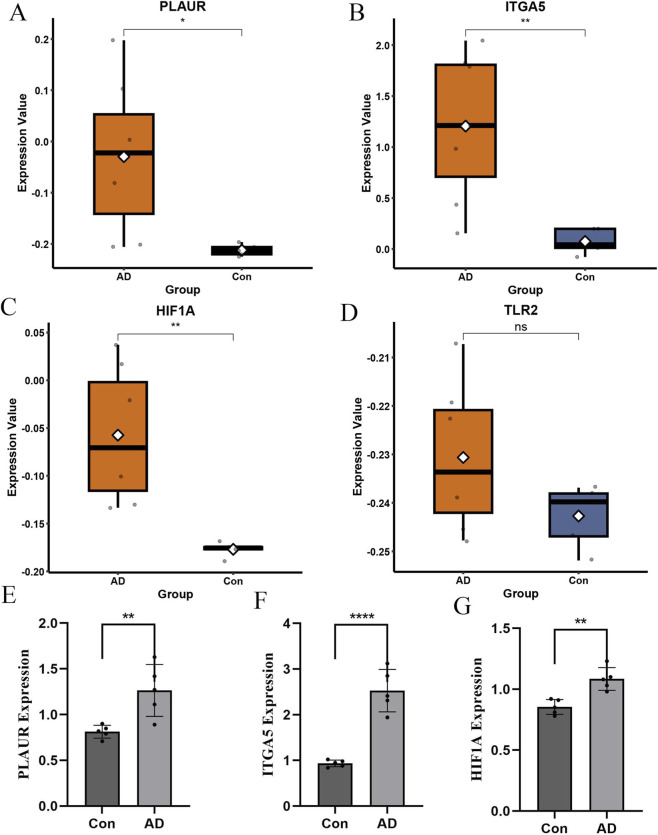
Validation of key candidate gene expression in external datasets and clinical samples. Expression levels of PLAUR **(A)**, ITGA5 **(B)**, HIF1A **(C)**, and TLR2 **(D)** in the external dataset. RT-qPCR validation of PLAUR **(E)**, ITGA5 **(F)**, and HIF1A **(G)** expression in clinical vascular tissue samples.

To further validate the robustness of the four identified key candidate genes, their expression patterns were examined in an independent transcriptomic dataset and clinical samples. In the external dataset GSE52093, including 7 aortic dissection samples and 5 control samples, differential expression analysis showed that PLAUR, ITGA5, and HIF1A were significantly upregulated in aortic dissection, whereas TLR2 did not show a significant difference between groups, as presented in Figures 7A–D. To further confirm these findings, quantitative real-time PCR was performed using vascular tissue samples collected from Henan Provincial Chest Hospital, including 10 specimens with 5 aortic dissection samples and 5 control samples obtained from patients undergoing coronary artery bypass grafting. The results demonstrated that PLAUR, ITGA5, and HIF1A were significantly increased in aortic dissection tissues compared with controls, and notably, TLR2 also exhibited a significant difference in the PCR validation, as shown in Figures 7E–G. Overall, these findings indicate that PLAUR, ITGA5, and HIF1A exhibit consistent and robust differential expression across datasets and experimental validation, demonstrating strong performance as diagnostic biomarkers in aortic dissection. To quantitatively evaluate the diagnostic performance of the validated key candidate genes, ROC curve analysis was conducted using expression data from the GSE52093 external validation dataset. As shown in Supplementary Figure S1, HIF1A demonstrated perfect discrimination between AD and control samples (AUC = 1.000), while ITGA5 and PLAUR both achieved AUC values of 0.943, indicating excellent discriminatory ability. These results provide preliminary quantitative support for the diagnostic potential of these three genes.

## 4 Discussion

This study integrates clinical data with multi-omics analyses to demonstrate that six composite inflammatory indices are significantly elevated in patients with aortic dissection (AD) and independently associated with disease risk. Furthermore, through combined bioinformatics and machine learning approaches, we identified HIF1A, ITGA5, PLAUR, and TLR2 as macrophage-associated genes, with experimental validation confirming the upregulation of HIF1A, ITGA5, and PLAUR in dissected aortic tissue.

Previous studies have mainly focused on the prognostic value of inflammatory indices in AD. For instance, the neutrophil-to-lymphocyte ratio (NLR) has been shown to predict in-hospital mortality in acute AD patients (Sbarouni et al., 2015; Lafci et al., 2014; Karakoyun et al., 2015), while the systemic immune-inflammation index (SII) has been associated with adverse aortic-related outcomes following thoracic endovascular repair (Su et al., 2022; Zhao et al., 2022; Chen et al., 2024). In contrast, our findings extend these observations by demonstrating that these indices also possess diagnostic value at presentation. Notably, restricted cubic spline analyses revealed distinct exposure-response patterns. AISI and SII showed approximately linear relationships with AD risk, whereas NLR, MLR, and SIRI exhibited threshold effects. This difference may reflect the broader biological scope captured by platelet-containing indices, which incorporate platelet-leukocyte interactions known to contribute to vascular inflammation and endothelial injury (Qin et al., 2016; Sahin and Sahin, 2026; Yang et al., 2025). By contrast, the plateau effect observed for NLR and MLR may be related to rapid neutrophil mobilization following intimal disruption, beyond which further increases provide limited incremental risk information.

Subgroup analyses further indicated that the associations of AISI, SII, and PHR with AD were stronger in individuals younger than 60 years. This pattern may be explained by the relatively lower burden of chronic inflammation in younger populations, allowing acute inflammatory signals to be more clearly detected (Wang et al., 2026; Shirakura et al., 2026). Additionally, the stronger association of SIRI in females suggests a potential role of sex hormones in modulating innate immune responses. Estrogen has been reported to influence neutrophil and monocyte function and trafficking, thereby shaping vascular inflammatory responses (Wagner et al., 2026; Jackson et al., 2026; Matsumoto et al., 2026; Utami et al., 2025). The use of generalized propensity score weighting strengthens the robustness of these findings by minimizing confounding from baseline differences such as hypertension and smoking.

At the cellular level, our single-cell RNA sequencing analysis demonstrated a marked expansion of macrophages in AD tissues, accompanied by substantial transcriptional reprogramming. This is consistent with previous studies showing enrichment of inflammatory macrophages in dissected aortas. Inoue et al. identified IL-1β producing macrophages derived from S100A8/9-positive monocytes as a key inflammatory component in acute AD (Inoue et al., 2024), while Zhang et al. reported increased macrophage infiltration and matrix metalloproteinase activation in type A AD (Zhang et al., 2023). By integrating single-cell and bulk transcriptomic data with curated inflammatory gene sets, our study refines candidate genes within a macrophage-specific context, thereby improving biological interpretability and reducing false-positive findings. A key question is how macrophage-driven transcriptional programs relate to elevated peripheral inflammatory indices. We propose that macrophage infiltration and activation in the dissected aortic wall-evidenced by single-cell analysis-drive local release of pro-inflammatory mediators (e.g., IL-1β, TNF-α, MMPs), promoting systemic leukocyte mobilization with increased neutrophils and monocytes and relative lymphocyte suppression, thereby elevating NLR, MLR, SIRI, and AISI. Hypoxia-induced HIF1A activation sustains macrophage inflammatory activity, while PLAUR and ITGA5 enhance migration and matrix degradation, amplifying vascular injury and immune recruitment. Concurrent endothelial disruption triggers platelet activation, linking these processes to SII and AISI, and reduced HDL-C anti-inflammatory capacity may underlie changes in PHR. Although direct correlations were not feasible due to separate cohorts, this framework provides a plausible link between local molecular alterations and systemic inflammatory signatures in AD.

Among the identified genes, HIF1A plays a central role in hypoxia-driven inflammation. Hypoxic microenvironments within the dissected aortic wall can activate HIF1A signaling, which in turn regulates macrophage metabolic reprogramming and inflammatory gene expression. Notably, HIF1A likely links hypoxia to inflammatory amplification in aortic dissection. HIF1A has been shown to transcriptionally regulate TLR2 and promote inflammatory responses under hypoxic conditions (Zhong et al., 2026; Li et al., 2025). PLAUR encodes the urokinase plasminogen activator receptor (uPAR), which is involved in extracellular matrix degradation and macrophage migration. Increased PLAUR expression has been reported in vascular remodeling and inflammatory conditions, where it facilitates matrix breakdown and cell infiltration (Zhu et al., 2026). ITGA5, a key integrin involved in cell-matrix adhesion, has also been implicated in aortic dissection through its role in extracellular matrix organization and inflammatory cell recruitment (Wang et al., 2025). Although not consistently significant across all datasets, TLR2 was robustly selected by machine learning models and upregulated in clinical samples. Its activation triggers NF-κB-dependent inflammatory and proteolytic pathways, further contributing to vascular inflammation and structural destabilization. Previous studies have demonstrated that TLR2 contributes to vascular inflammation and remodeling in response to endogenous danger signals (Singh et al., 2026; Schettino et al., 2026).

Several limitations should be acknowledged. This study has several limitations. The retrospective, single-center design may introduce residual confounding despite adjustment. The RT-qPCR validation cohort was small, and larger studies are needed for confirmation. The single-cell analyses relied on public datasets with limited sample sizes and potential batch effects. Moreover, functional experiments are required to establish causal links between the identified genes and AD pathogenesis. Importantly, the case-control design measured inflammatory indices at the time of diagnosis rather than prior to onset, precluding temporal inference. Elevated indices may therefore reflect acute systemic inflammation secondary to dissection (e.g., immune activation following intimal disruption and medial injury) rather than pre-existing susceptibility. Accordingly, the logistic regression results should be interpreted as cross-sectional odds ratios rather than prospective risk estimates. Future prospective studies with serial measurements before and after AD onset are warranted to clarify temporal and causal relationships.

## 5 Conclusion

In summary, this study identified four inflammation-related genes associated with aortic dissection through integrated bioinformatic and machine learning analyses, with preliminary support from RT-qPCR validation. These findings provide candidate molecular signatures and generate testable hypotheses regarding the inflammatory mechanisms underlying aortic dissection. However, given the exploratory nature of this study and the limited sample size of experimental validation, further validation in larger independent cohorts, together with protein-level and prospective studies, is required to determine their biological robustness and potential translational relevance.

## Data Availability

The datasets presented in this study can be found in online repositories. The names of the repository/repositories and accession number(s) can be found in the article/Supplementary Material.
