# Supplementary material for: Integrated clinical and multi-omics analysis links composite inflammatory indices to macrophage-associated molecular programs in aortic dissection
Source: Front Genet. 2026 May 21;17:1840880. doi: 10.3389/fgene.2026.1840880 (PMC13233053; doi:10.3389/fgene.2026.1840880)
Supplement: Supplementary file 1 [file DataSheet1.pdf]

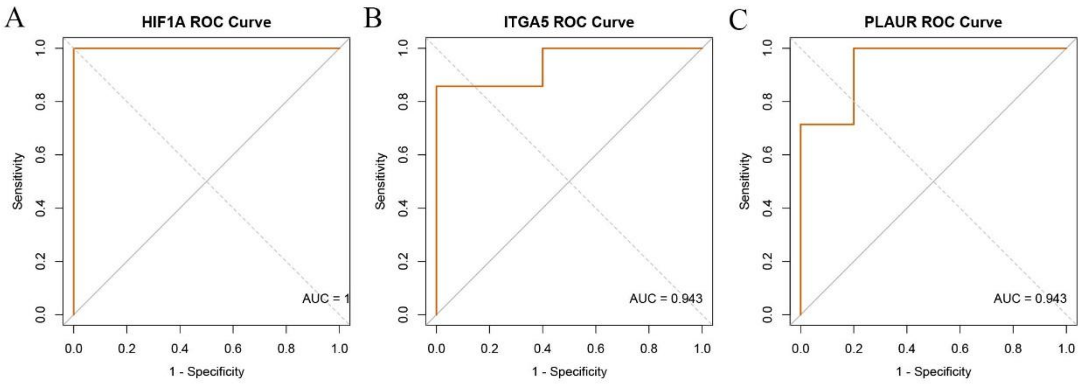

Figure S1. ROC curve analysis of hub genes in the external validation dataset (GSE52093). ROC curves for HIF1A (A), ITGA5 (B), and PLAUR (C) based on gene expression data from 7 AD samples and 5 control samples. AUC values are displayed within each panel. The diagonal dashed line represents chance-level discrimination (AUC = 0.5).
